# Supplementary material for: Mechanisms and interventions promoting healthy frontostriatal dynamics in obsessive-compulsive disorder
Source: Nat Commun. 2025 Aug 11;16:7400. doi: 10.1038/s41467-025-62190-2 (PMC12340154; doi:10.1038/s41467-025-62190-2)
Supplement: Supplementary file 1 — Supplementary Information [file 41467_2025_62190_MOESM1_ESM.pdf]

Mechanisms and interventions promoting healthy frontostriatal dynamics in obsessive-compulsive disorder

Supplementary Materials

Sébastien Naze[1], Luke J. Hearne[1], Paula Sanz-Leon[1,2], Conor Robinson[1], Caitlin V. Hall[1], Saurabh Sonkusare[1], Bjorn Burgher[1], Andrew Zalesky[3,4], James A. Roberts[1], and Luca Cocchi[1,5]

[1]QIMR Berghofer Medical Research Institute, Brisbane, Queensland, Australia.
[2]School of Physics, Faculty of Science, The University of Sydney, Sydney, New South Wales, Australia.
[3]Department of Psychiatry, The University of Melbourne, Melbourne, Victoria, Australia.
[4]Department of Biomedical Engineering, The University of Melbourne, Melbourne, Victoria, Australia.
[5]School of Biomedical Sciences, Faculty of Health, Medicine and Behavioural Sciences, University of Queensland, Brisbane, Queensland, Australia.

Contents

Supplementary Figure 1: Comparison of nucleus accumbens (NAcc) functional connectivity maps between Harrison et al. (2009) and Naze at al. (2023) 2
Supplementary Figure 2: Comparison of dorsal putamen (dPut) functional connectivity maps between Harrison et al. (2009) and Naze at al. (2023) 3
Supplementary Figure 3: Empirical *vs.* simulated frontostriatal functional connectivity . . 4
Supplementary Figure 4: Model extension with thalamic nuclei and relaxed priors confirms changes in neural coupling in and within the OCD ventromedial and dorsolateral circuits but fails to improve accuracy 5
Supplementary Figure 5: Generation of virtual cohorts and evaluation of virtual interventions. 6
Supplementary Figure 6: Associations between parameter values and intervention efficacies 7
Supplementary Figure 7: Individualised intervention framework 8
Supplementary Figure 8: Medicated versus unmedicated OCD subjects do not display clear differences in frontostriatal parameters and functional connectivity across time 9
Supplementary Figure 9: Absence of association between improvement in frontostriatal functional connectivity and other comorbid clinical measures 10
Supplementary Figure 10: Diagram of the basal ganglia circuitry and network simplifications 11
Supplementary Table 1: Posterior distributions' statistics 12
Supplementary Table 2: Default model parameters 12

Supplementary Section I: Dynamical system analysis of the two-population model 13
Supplementary Figure 11: State space of the simplified two regions circuit 13
Supplementary Figure 12: Bifurcation diagrams of the simplified two regions circuit . . . 14

Supplementary Section II: Parameter estimation and optimization 15
1 Bayesian framework 15
2 Approximate Bayesian Computation (ABC) 15
3 Sequential Monte-Carlo: ABC-SMC 15
Supplementary Figure 13: Convergence of the sequential optimization algorithm 16

Supplementary References 17

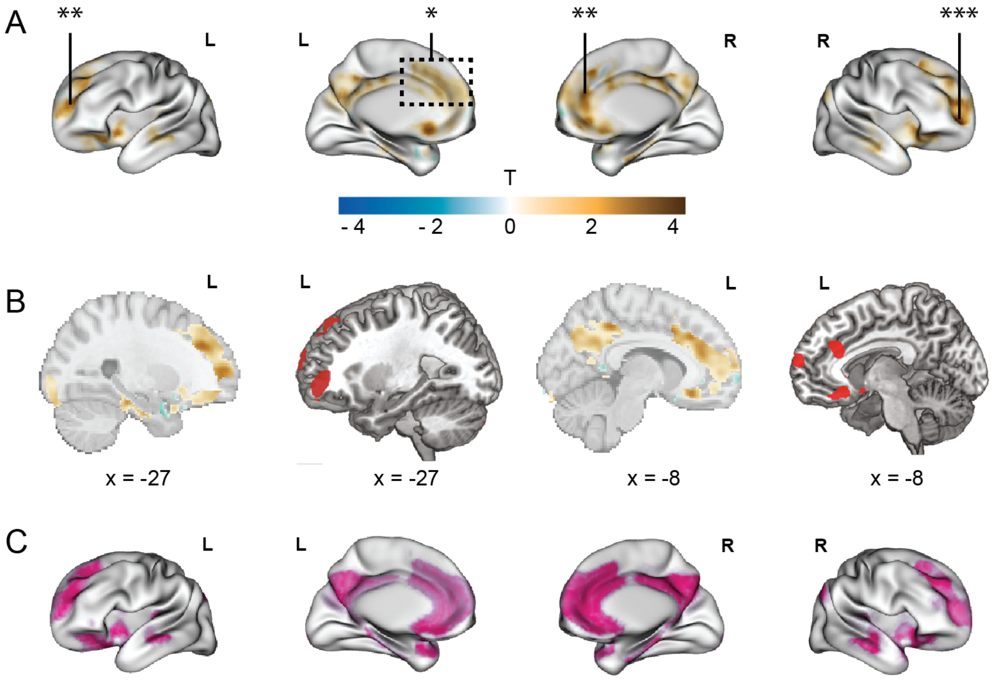

Supplementary Figure 1: Comparison of nucleus accumbens (NAcc) functional connectivity maps between Harrison et al. (2009) [1] and Naze at al. (2023) [2] A. Analyses on the dataset adopted for the current study showed that NAcc-frontal hyperconnectivity at rest is highest in the right medial orbitofrontal cortex (OFC) in OCD compared to controls ($|T| > 4.2$, $***p_{FWE} < 0.05$, at MNI x=28, y=60, z=-2). Adopting a more lenient threshold revealed higher functional connectivity in OCD also in the left medial OFC ($**p < 0.001$; uncorrected, x=-24, y=60, z=8) and the anterior cingulate cortex (ACC, $*p < 0.01$; x=-6, y=38, z=22). B. Side-by-side comparisons between the left hemisphere hyperconnectivity in OCD relative to controls observed in Harrison et al. [1]($p_{FDR} < 0.05$, in red) and Naze et al. [2] ($p < 0.001$, same color-code as panel A) show strong spatial overlap. C. Cortical regions showing significant functional connectivity with the NAcc ($p_{FWE} < 0.05$, see Naze et al. [2]). These maps were obtained using a large normative sample (HCP 1080 [3]). Results highlight that the frontal clusters showing higher functional connectivity between the NAcc and the frontal cortex in OCD across the two studies (panel A and B) map onto the same functional circuit. Coordinates are in the MNI space. Images from Harrison et al. (2009) used with permissions.

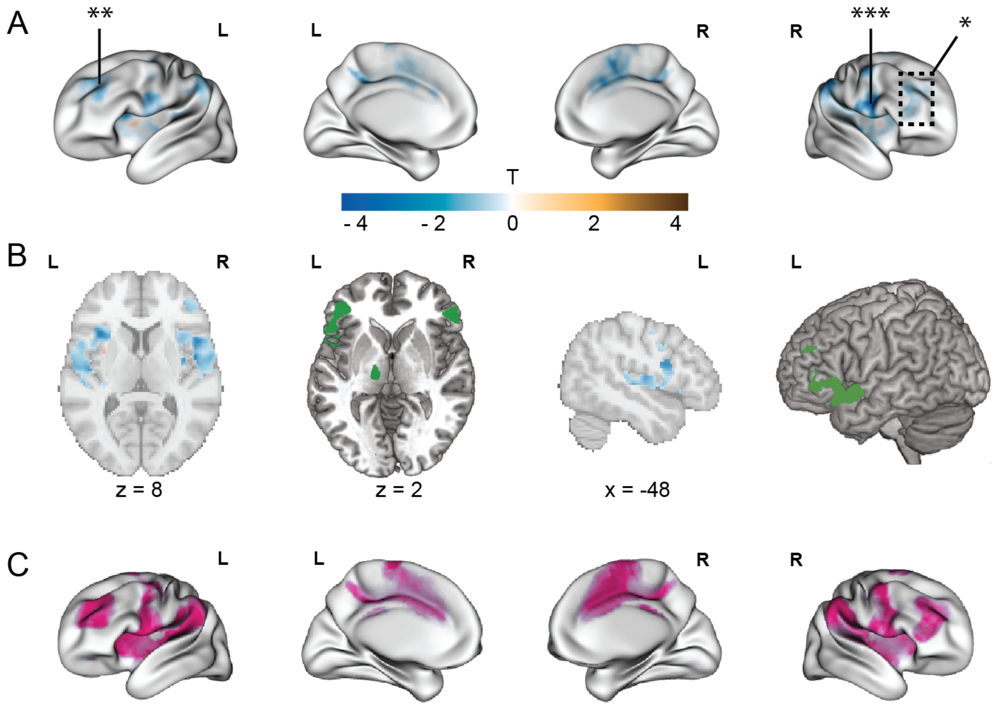

Supplementary Figure 2: Comparison of dorsal putamen (dPut) functional connectivity maps between Harrison et al. (2009) [1] and Naze at al. (2023) [2] A. Results from analyses of our dataset (Naze et al., 2023) show hypoconnectivity between the dorsal putamen and the right lateral prefrontal cortex in OCD relative to controls ($|T| > 3.5$, ***$p_{FWE} < 0.05$, MNI x=53, y=13, z=19). Hypoconnectivity between the putamen and more anterior frontal cluster is also observed when more lenient (uncorrected) statistical thresholds were applied (**$p < 0.001$ for the cluster in the left hemisphere (x=-38, y=44, z=26) and *$p < 0.005$ for the cluster in the right hemisphere (x=42, y=38, z=26)). B. Side-by-side comparison between the results of Harrison et al. (2009) [1] (green, $p < 0.001$) and Naze et al. (2023) [2] color-code as in panel A, highlight a strong overlap between the frontal clusters showing reduced resting-state functional connectivity with the dorsal putamen. C. Cortical regions showing significant ($p_{FWE} < 0.05$) functional connectivity with the dorsal putamen. These maps are obtained using a large normative sample (HCP 1080 [3]). The results highlight that the frontal clusters showing reduced functional connectivity between the dorsal putamen and the frontal cortex in OCD across the two studies (panels A and B) map onto the same functional circuit. Coordinates are in the MNI space. Images from Harrison et al. (2009) used with permissions.

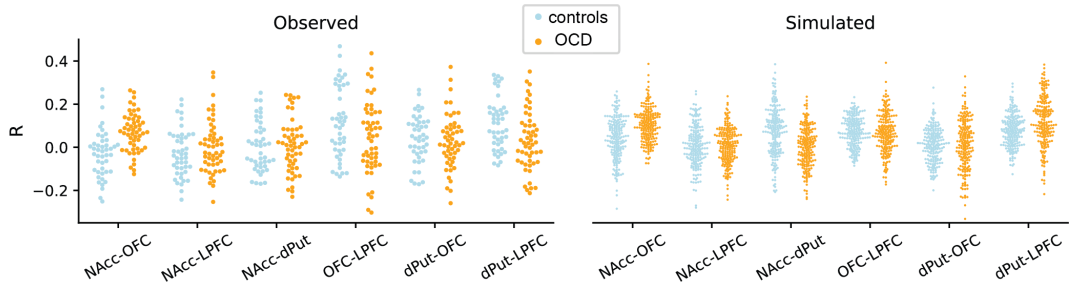

Supplementary Figure 3: Observed (fMRI) and simulated (model) functional connectivity values (Pearson correlation coefficient, R) across the frontostriatal connections of interest. The model parameters from the simulated data are drawn from posterior distributions presented in Figure 2B (main text). Sample size $n = 64$.

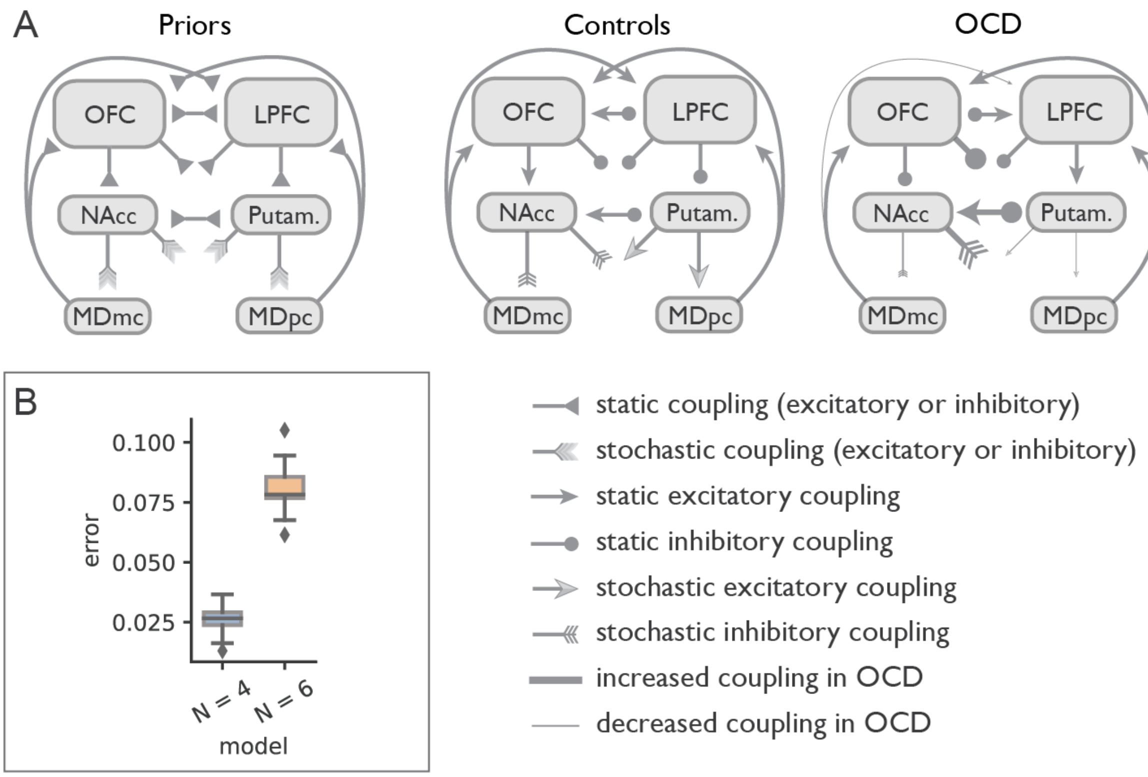

Supplementary Figure 4: A. Illustration of the fronto-striato-thalamo-cortical model with all cross-pathway (but the thalamo-thalamic [4]) connections. For this model, we removed priors on the nature of cortical and thalamic efferents being only excitatory. Differences in couplings between controls and OCD are indicated by a change in arrow size (magnitude) and arrow ends (sign) as indicated in the legend. B. The root mean square error of the fitting to empirical data after inference (i.e., the inverse of model evidence) for the original model (main text, N = 4 regions, blue) and the relaxed model with thalamic nodes (N = 6 regions, orange). Sample size $n = 20$ (i.e. 20 cohorts of 50 virtual subjects, compared to the empirical data of 1 cohort of 50 real subjects, resulting in 20 error values). Boxes indicate upper and lower quartiles with whiskers extending to 1.5 times the inter-quartile range. Diamonds denote outliers. OFC: orbitofrontal cortex, LPFC: lateral prefrontal cortex, NAcc: nucleus accumbens, Putam.: dorsal putamen, MDmc/MDpc: magnocellular/parvocellular mediolateral thalamus.

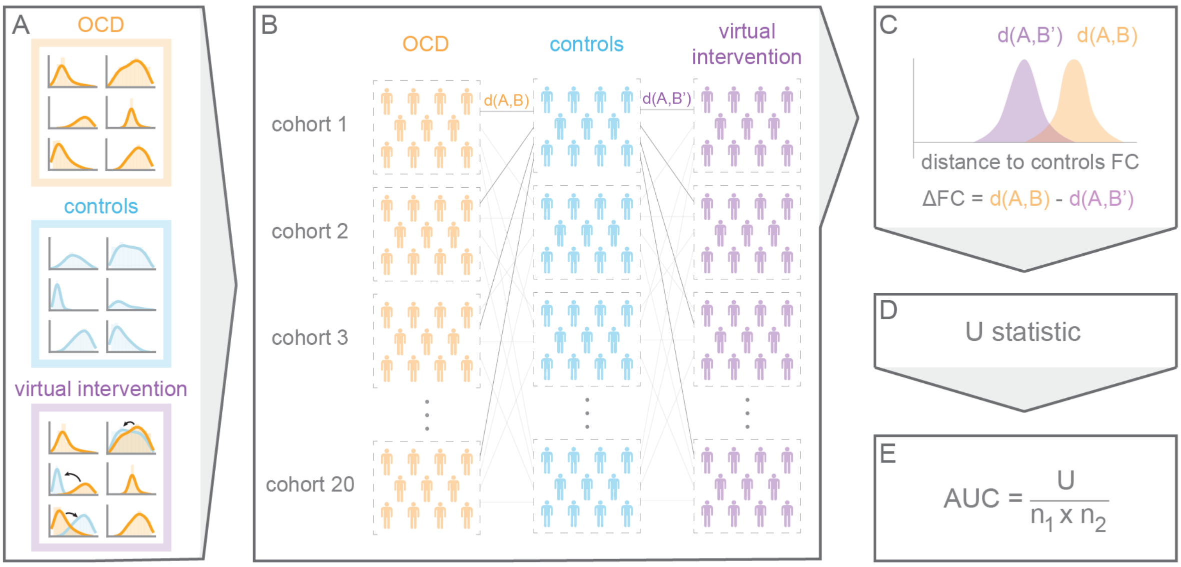

Supplementary Figure 5: Generation of virtual cohorts and evaluation of virtual interventions. A. Parameters are drawn from OCD (orange) and control (blue) posterior distributions to create 2000 reference virtual subjects, 1000 in each group. Virtual interventions are modelled by drawing from the reference control group distributions for the parameters targeted by the intervention and drawing from the reference distributions of the OCD group for parameters not targeted by the intervention. 1000 virtual subjects are generated to create the virtual intervention cohorts. B. For each group, the 1000 virtual subjects are separated into 20 cohorts of 50 subjects. We computed functional connectivity (FC) distances between all controls and OCD cohorts ($d(A, B)$; i.e., reference); and all controls and virtual interventions cohorts ($d(A, B')$; i.e., intervention). C. The distribution of FC distances between reference (orange) and intervention (purple) is compared (decrease in the distance implies functional improvement). D. The efficacy of the intervention is statistically quantified using a Mann-Whitney U test between reference and intervention distributions. E. Normalization of the U statistic by the number of samples leads to the AUC for which scores above 0.5 denote functional improvement.

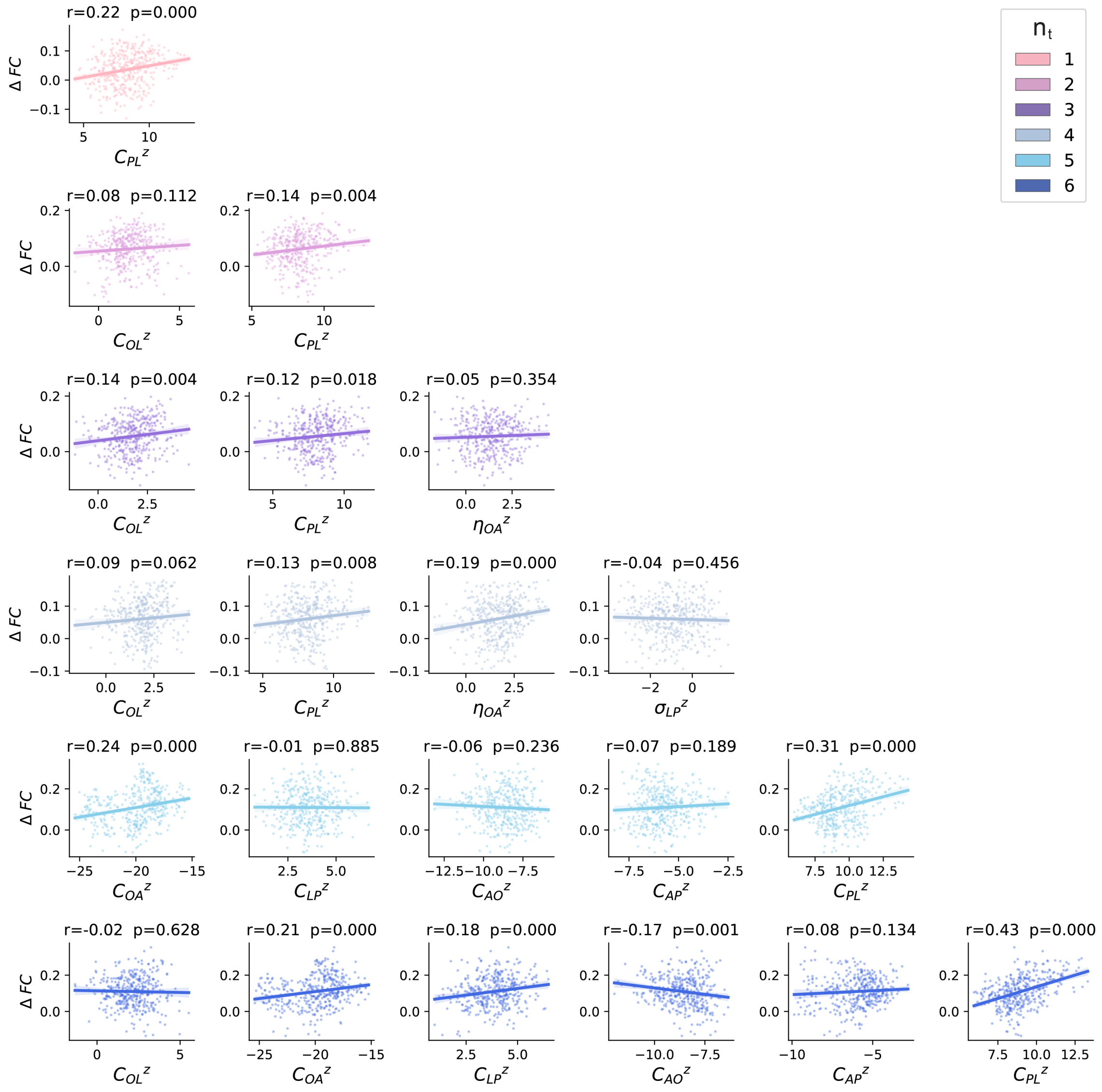

Supplementary Figure 6: Associations between parameter values and intervention efficacies. Z-score normalized parameter values (θ^z, where θ represents the parameter name; x-axis) against intervention efficacy (difference in distance to healthy controls in functional connectivity space, ΔFC; y-axis). Numbers of targets n_t are indicated by the same color code as main text's Figure 4. Only the best intervention (highest AUC) is shown for each number of targets. Sample size $n = 400$.

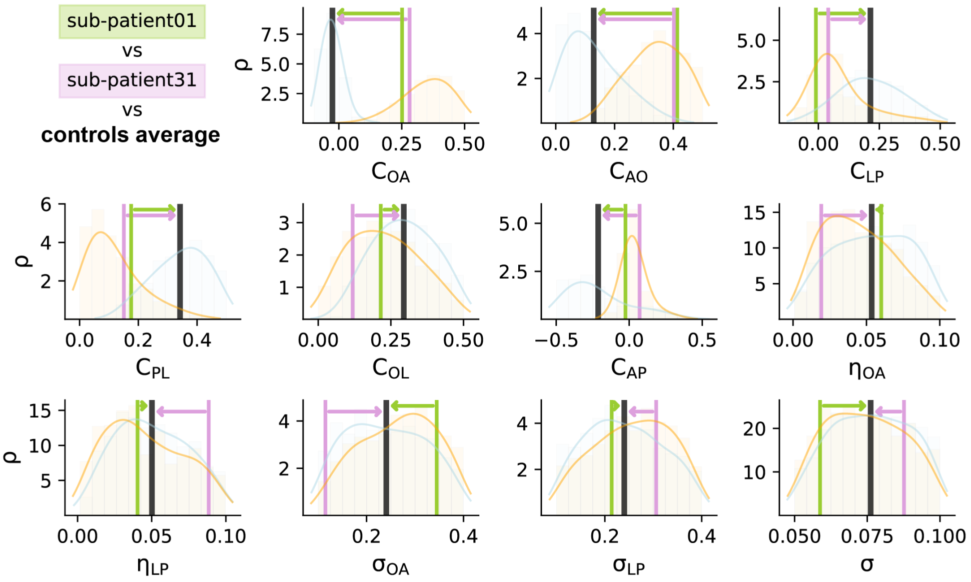

Supplementary Figure 7: Digital twin parameters (vertical coloured lines; green: OCD #01, purple: OCD #31) are displayed alongside the control group's average (black vertical line). Arrows indicate the direction (arrowhead) and amplitude (vector length) of the targeted changes in specific neural parameters to restore healthy neural dynamics at the individual level. Background distributions are posteriors of OCD (orange) and controls (blue) as per Figure 3 (main text).

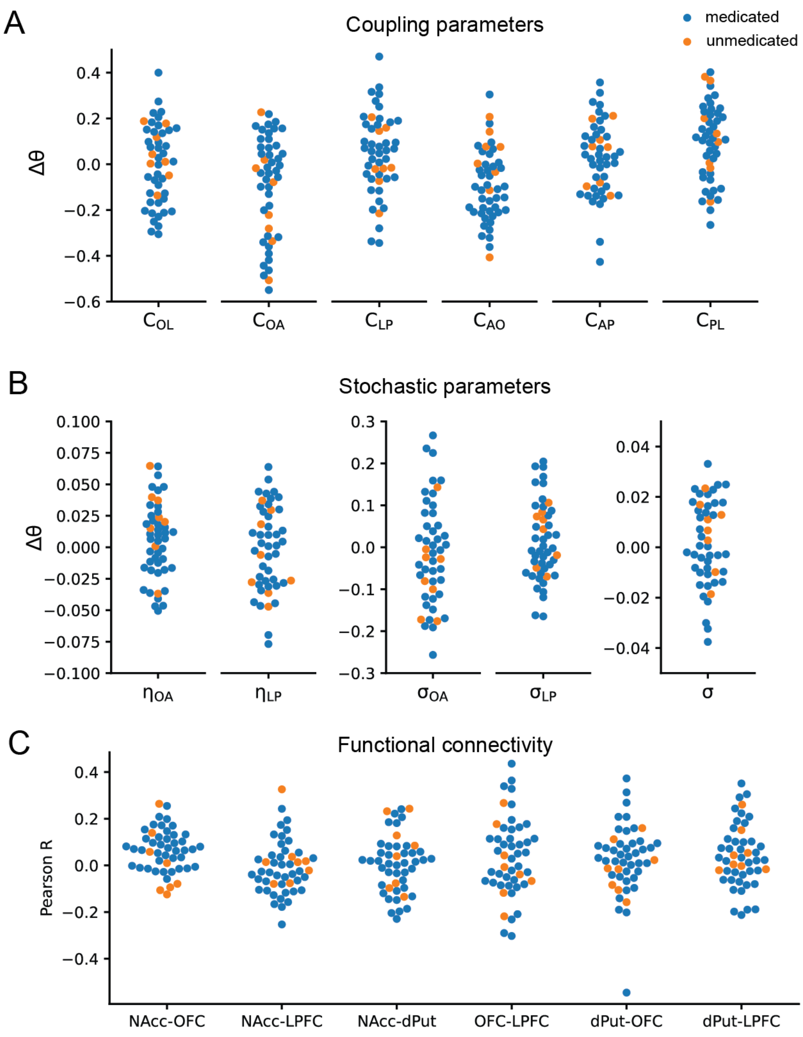

Supplementary Figure 8: Medicated versus unmedicated OCD subjects do not display clear differences in frontostriatal parameters and functional connectivity across time. A-B. Parameter differences ($\Delta\theta$) in OCD digital twins between initial and follow-up assessments. Medicated (blue) and unmedicated (orange) subjects do not show statistically significant differences in any model parameter. C. Frontostriatal functional connectivity between medicated (blue) and unmedicated (orange) OCD subjects. NAcc: nucleus accumbens, dPut: dorsal putamen, OFC: orbitofrontal cortex, LPFC: lateral prefrontal cortex. Sample size $n = 48$.

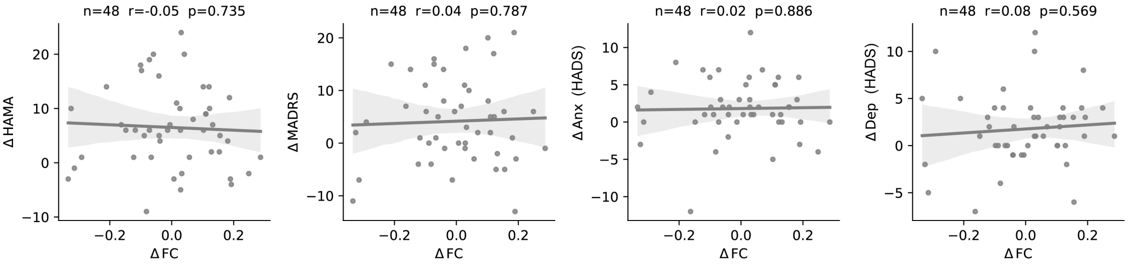

Supplementary Figure 9: Absence of association between improvement in frontostriatal functional connectivity and other comorbid clinical measures. Improvement in frontostriatal functional connectivity (ΔFC) is calculated as the difference of FC distances (in FC space across ventromedial and dorsolateral circuits) to mean healthy controls' FC for each OCD subjects (at initial minus follow-up values). Likewise, improvements in clinical measures are calculated as the difference between symptoms at baseline and at follow-up. HAMA: Hamilton Anxiety Rating Scale; MADRS: Montgomery-Asberg Depression rating scale; HADS: Hospital Anxiety (Anx) and Depression (Dep) scale. Gray shaded area denote 95% confidence interval estimated by bootstrapping method with 1000 resamples.

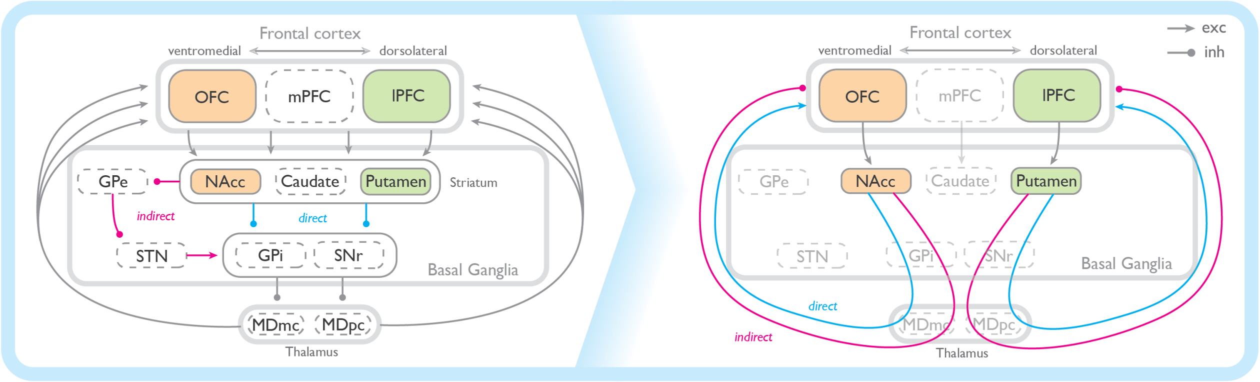

Supplementary Figure 10: Diagram of the basal ganglia circuitry and network simplifications. The frontal cortex can be coarsely functionally partitioned into a ventromedial (affective) circuit and a dorsolateral (cognitive) circuit [5, 6]. The basal ganglia encompass the striatum, the globus pallidus (GP), the substantia nigra (SN) and the subthalamic nucleus (STN) (left panel). The net effect of the basal ganglia projections to the cortex via the thalamus has been conceptualized as the direct (disinhibitory, blue) and indirect (inhibitory, pink) pathways. Marked changes in functional connectivity in the ventromedial circuit (in orange) and the dorsolateral circuit (in green) were previously identified in OCD using neuroimaging [2]. The whole system (left panel) is simplified in our network model (right panel), where striato-pallido-thalamo-cortical projections and the interplay between direct and indirect pathways are reduced to a dynamic coupling with both excitatory and inhibitory effects. OFC: orbitofrontal cortex, mPFC/lPFC: medial / lateral prefrontal cortex, NAcc: nucleus accumbens, GPe/GPi: globus pallidus externa/interna, SNr: substantia nigra pars reticulata, MDmc/MDpc: magnocellular/parvocellular mediolateral thalamus.

**Parameter (θ)**	**Normality**	U	p	p_{FWE}	**Cohen's d**
C_{OL}	False	665365	1.51997e-37	2.12796e-36	0.611949
C_{OA}	False	472	<2.2251e-308	<2.2251e-308	-5.15583
C_{LO}	False	514146	0.273327	3.82658	0.0399174
C_{LP}	False	800665	6.50742e-120	9.11038e-119	1.12387
C_{AO}	False	66912	1.32766e-246	1.85873e-245	-2.26136
C_{AP}	False	158316	2.79392e-154	3.91148e-153	-1.53297
C_{PL}	False	942352	3.57043e-257	4.99861e-256	2.41961
C_{PA}	False	528129	0.0293852	0.411393	0.409623
G	False	517229	0.182145	2.55003	0.0658576
η_{OA}	False	608626	4.03104e-17	5.64346e-16	0.387891
η_{LP}	False	556215	1.34123e-05	0.000187772	0.184019
σ	False	539045	0.00249771	0.034968	0.135214
σ_{OA}	False	417249	1.47224e-10	2.06114e-09	-0.291643
σ_{LP}	False	434998	4.81011e-07	6.73416e-06	-0.222404

Supplementary Table 1: Posterior distribution statistics. Healthy controls and OCD posterior distributions of parameters are compared after fitting the model to empirical data, using a two-sided Mann-Whitney U test. P-values are reported uncorrected (p) and with multiple-comparison correction (family-wise error, p_{FWE}). Values are bounded to minimal and maximal floating point values. Effect size is reported as Cohen's d. Sample size $n = 1000$.

**Parameter**	**Value**	**(unit) Interpretation**
a	270	(n/C) slope of the transfer function
b	108	(Hz) offset of the transfer function
d	0.154	(s) decay of the transfer function
G	2.5	(N/A) global coupling gain of the system
J_N	0.2609	(nA) synaptic scaling factor
I_0	0.3	(nA) external input
τ_S	0.1	(s) timescale of the local population activity
γ	0.641	(Hz) timescale of the coupled activity
w	0.9	(N/A) recurrent excitation factor
σ	0.001	(nA) noise amplitude

Supplementary Table 2: Default model parameters

Supplementary Section I: Dynamical system analysis of the two-population model

We performed a numerical analysis of the dynamics of the simplified circuit composed of one frontal (S_1) and one striatal (S_2) region. This system is modelled using two coupled reduced Wong-Wang models [7], and its dynamics are analysed using PyDSTool (v1.23.5) [8]. Supplementary Figure 11 shows the state space of the system for a range of coupling parameters (fronto-striatal coupling C_{21} and striato-frontal coupling C_{12}). We observe that when at least one of the populations is inhibitory (i.e. $C_{12} < 0$ or $C_{21} < 0$) are population indices), the system displays only a single stable fixed point near $(S_1, S_2) = (0, 0)$. This indicates that, regardless of the current state of the system, both populations will eventually come back to their baseline activity after a perturbation is introduced in the state variables [9].

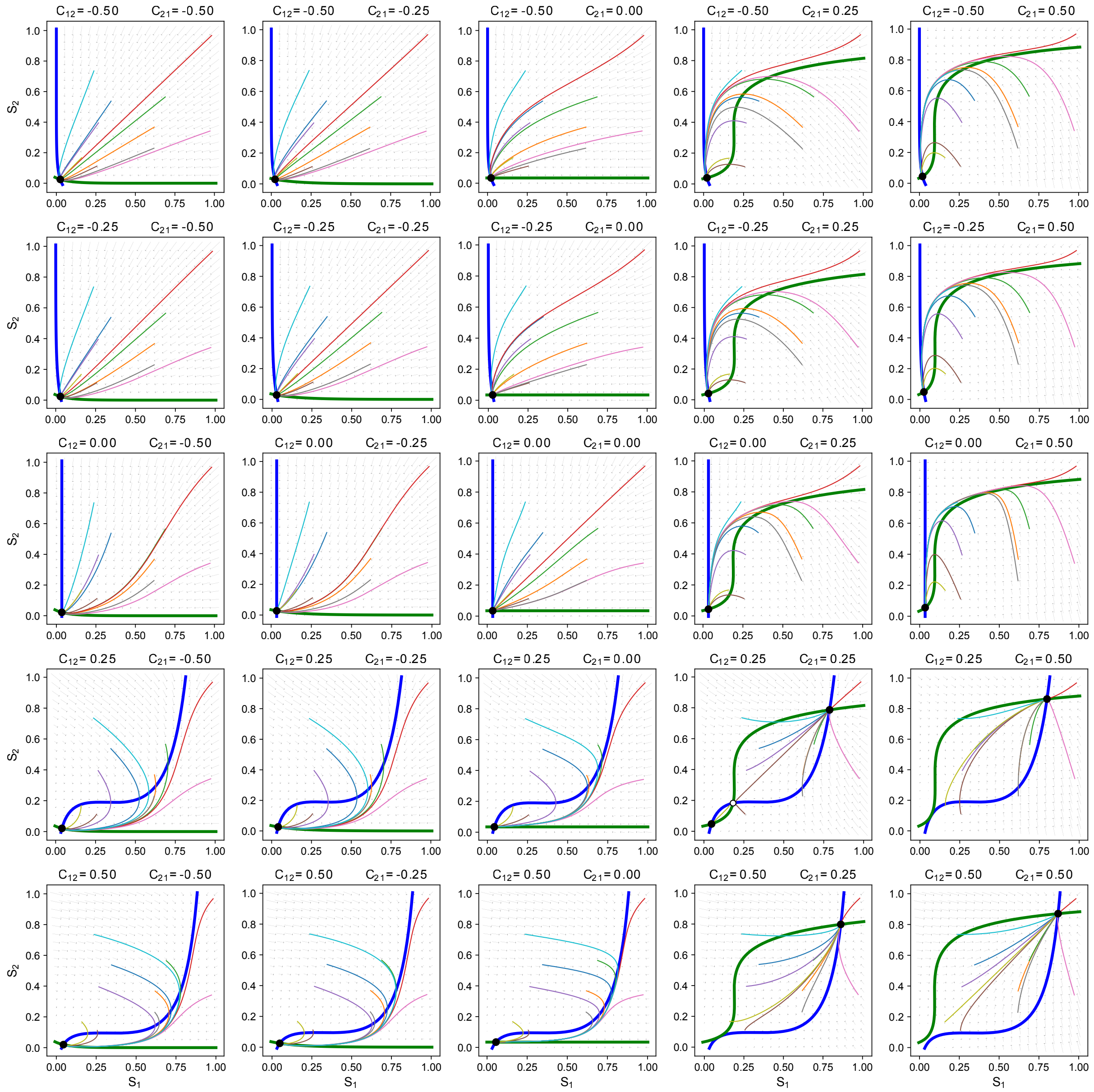

Supplementary Figure 11: State space of the system of the simplified circuit composed of one striatal and one frontal region. S_1 and S_2 are the state variables (population firing rates) of the Reduced Wong-Wang models, C_{ij} denotes the coupling strength from population j to population i. Blue and green thicker lines are the nullclines (i.e., the solution of each population at equilibrium: $dS_1/dt = 0$ and $dS_2/dt = 0$); thinner lines are trajectories computed using 10 random initial conditions $S_1, S_2 \in [0, 1]$. Black and while circles are stable and unstable fixed points, respectively. Vector fields denoted by grey arrows in the background indicate activity flow.

When both populations become excitatory ($C_{12} > 0$ and $C_{21} > 0$, lower right panels), another stable fixed point appears in a state where both populations are highly active ($(S_1, S_2) \sim (1, 1)$), with a saddle point in the middle (near $(S_1, S_2) \sim (0.2, 0.2)$ in panel with $C_{12} = 0.25$ and $C_{21} = 0.25$). This indicates that the system went through a bifurcation (i.e. a state transition) and is now bistable. This parameter regime is of special interest because more complex dynamics can be observed when perturbations or random fluctuations knock the system back and forth between high-activity and low-activity states, the latter representing a baseline activity. Finally, when both populations are strongly coupled through mutual excitation, $(C_{12}, C_{21}) \sim (0.5, 0.5)$ and above, the system displays a single stable fixed point in a state of high activity in both populations.

Next, we fix one of the inter-population coupling and use the other as control parameter to produce bifurcation diagrams of the coupled system. A bifurcation diagram indicates how fixed points of the system (i.e., the attractor landscape), changes as the control parameter is continuously shifted. Given that the dynamically richer regimes, where bistability occurs, are located in the lower right quadrant of the state space matrix presented in Supplementary Figure 11 (where at least one population is excitatory), we investigate the bifurcation structure of the system when the excitation of one population is discretized and the other population coupling serves as control parameter (continuously changing from strongly inhibitory to strongly excitatory). Supplementary Figure 12 shows how the bifurcation diagrams of the two-population system change in response to a gradual increase of the excitatory coupling C_{21}. Numerical analysis used forward and backward paths from fixed points, with adaptive step size between 10^{-6} and 10^{-5}).

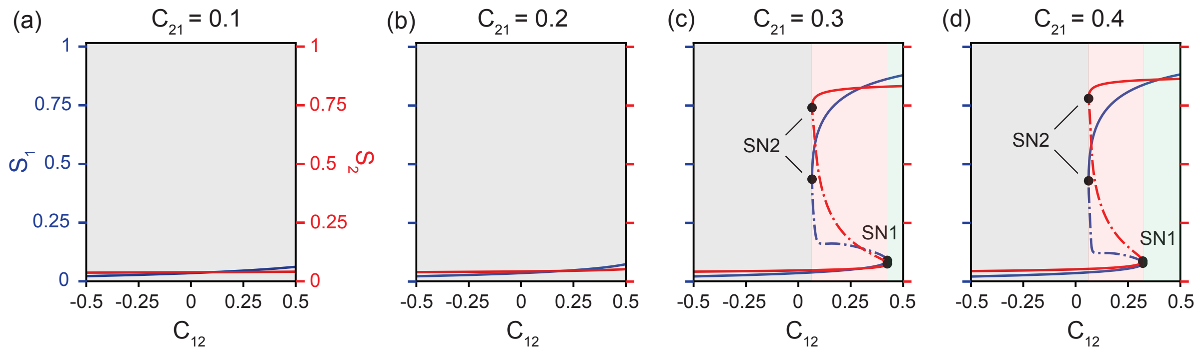

Supplementary Figure 12: Bifurcation diagrams of the simplified circuit composed of one striatal and one frontal region for increasing values of coupling parameters (a-d). S_1 (blue) and S_2 (red) are the state variables (population average synaptic gating) of two coupled Reduced Wong-Wang models, C_{ij} denotes the coupling strength from population j to population i. Note that C_{12} is changed continuously between -0.5 and 0.5 and serves as control parameter, while C_{21} takes discretized values. Lines denote equilibrium point curves (plain: stable, dash: unstable); SN: saddle-node points. Background: mono-stable low firing (gray), bistable (orange), mono-stable high firing (green).

We observe that for low values of C_{21} coupling, the dynamics lay in a regime where both populations have low firing rates (Suppl. Fig. 12a-b, gray background). For stronger excitatory coupling ($C_{21} > 0.2$), a regime of bistability emerges between two saddle-node bifurcations occuring at $C_{12} \leq 0.4$ (SN1) and $C_{12} \geq 0.05$ (SN2), where both high and low firing rates can be present (Suppl. Fig. 12c-d, orange background). Above this bistability region, only a state of high firing in both populations can be observed (Fig. 12c-d, green background).

Supplementary Section II: Parameter estimation and optimization

1 Bayesian framework

Bayesian optimization is rooted in the Bayesian inference framework, which infers parameter distributions θ from data points $\mathcal{D}$:

$$P(\theta|\mathcal{D}) = \frac{P(\mathcal{D}|\theta)\, P(\theta)}{P(\mathcal{D})}, \tag{1}$$

where $P(\theta|\mathcal{D})$ is the *posterior*, $P(\mathcal{D}|\theta)$ the *likelihood*, $P(\theta)$ the *prior*, and $P(\mathcal{D})$ the *evidence*.

The aim of the optimization is to find the posterior distribution that best explains the observations given the prior and the likelihood. In many areas of medicine and biology, observations (*evidence*) are extracted from experiments conducted under specific constraints (*likelihood function*) to test a hypothesis (via an experimental design) given some assumptions (*prior*). The collection and analysis of experimental data allow us to create and update models of the underlying system tested. When these models are formulated mathematically, we can use computer simulations to create new *synthetic* data, sidestepping the difficulty of calculating the likelihood function [10].

Algorithms that compute the posterior distribution without knowing the true likelihood but using the approximation of the likelihood through simulations are called Approximate Bayesian Computation (ABC) methods [11].

2 Approximate Bayesian Computation (ABC)

ABC methods sample parameters from the prior distribution $P(\theta)$ and simulate synthetic data $\hat{\mathcal{D}}$ which is compared to the observed data $\mathcal{D}$ using a distance function $\rho(\cdot)$. In the simplest form, if the resulting distance is under the acceptance threshold ϵ, the sampled set of parameter values $\hat{\theta}$ is saved, otherwise it is discarded. New parameter values are sampled from the prior distribution until reaching a number N of accepted samples. Each vector of accepted parameters represents an independent draw from the posterior distribution. This is formalized by the following equation:

$$P(\theta|\mathcal{D}) \propto P(\theta) \int \left(\epsilon - \rho(\mathcal{D}, \hat{\mathcal{D}})\right)\, d\theta. \tag{2}$$

Note that in comparison to Eq. (1), $P(\mathcal{D})$ can be omitted because it only serves as a constant scaling factor [12].

Such methods are called *importance sampling* and are computationally expensive, with the simplest one described above referred as Monte-Carlo sampling. More efficient algorithms have been developed to reach faster convergence towards the optimal posterior distribution by drawing new parameters based on previously accepted parameters using Markov chains, and are known as Markov-Chain Monte-Carlo methods (MCMC).

In MCMC methods, rather than independently sampling from the prior distribution $P(\theta)$ until reaching N accepted particles, new parameters are drawn from an intermediate distribution $\pi(\theta)$ generated using a multivariate Gaussian perturbation kernel $\mathcal{K}(\theta \to \hat{\theta})$ applied to previously accepted particles θ to create $\hat{\theta}$ [12, 13]. Current versions of such algorithms iterate several times over this sampling process with more and more stringent acceptance conditions to reach faster and more accurate estimates of the posterior distribution [11], they are therefore called *sequential*.

3 Sequential Monte-Carlo: ABC-SMC

In the Sequential Monte Carlo algorithm, parameters of accepted particles from one generation at time t are used to create the intermediate distribution $\pi_t(\theta)$ from which new parameters are sampled to create the next generation at time $t+1$. This process is called *sequential importance sampling* [14]. Weights are attributed to each new accepted sample parameter $\theta_t^{(i)}$ based on the ratio between its expectation from the intermediate distribution π_t and a *proposal distribution* η_t, which is a criterion comparing novelty with respect to the previous generation:

$$w_t(\theta_t^{(i)}) = \frac{\pi_t(\theta_t^{(i)})}{\eta_t(\theta_t^{(i)})}. \tag{3}$$

where superscript i only refers to the incrementing index of the accepted particles at each generation.

The proposal distribution is computed using the transition kernel $\kappa_t(\theta_{t-1}, \theta_t^{(i)})$ giving the probability of sampling $\theta_t^{(i)}$ from the previous generation of accepted parameters θ_{t-1}:

$$\eta_t(\theta_t^{(i)}) = \int \pi_{t-1}(\theta_t^{(i)})\, \kappa_t(\theta_{t-1}, \theta_t^{(i)})\, d\theta_{t-1}, \tag{4}$$

$$= \int \eta_{t-1}(\theta_t^{(i)})\, w_{t-1}(\theta_{t-1})\, \kappa_t(\theta_{t-1}, \theta_t^{(i)})\, d\theta_{t-1}. \tag{5}$$

This weighting mechanism through w_t effectively balances the exploration versus exploitation trade-off from one generation to the next. Note that at $t = 0$, both the intermediate and the proposal distribution are drawn from the prior distribution: $\pi_0 = \eta_0 = P(\theta)$, and all weights are set to $w_0 = 1/N$.

Concurrently, the acceptance threshold (or tolerance) ϵ_t is decreased from one generation to the next until it reaches its target value. This results in a faster estimation of the posterior distribution, starting with high tolerance (exploration) and finishing with low tolerance (exploitation).

Supplementary Figure 13 shows the evolution of this tolerance threshold ϵ and the weights w associated with each accepted particle for each generation of the sequential algorithm. After 10 generations, the tolerance ϵ reaches approximately the same value for optimization against healthy controls and OCD subjects, indicating comparable goodness of fit. The observed weights indicate a successful optimization without particle degeneracy, i.e. the over-weighting of only a few particles [15].

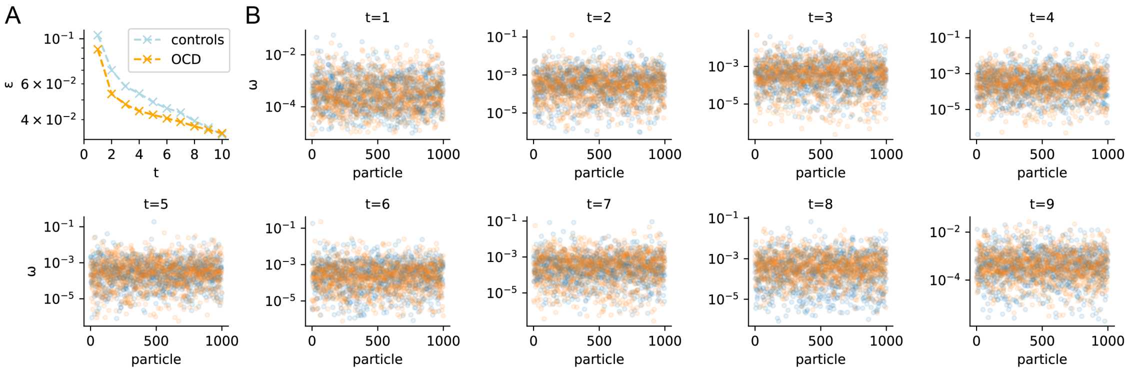

Supplementary Figure 13: Convergence of the sequential optimization algorithm. **A.** Adaptable tolerance threshold ϵ for which a particle (i.e., a run of simulations with a given parameter set) is *accepted*. **B.** Weights w attributed to accepted particles at each generation t. Same color scheme as in **A**.

Supplementary References

1. Harrison, B. J. *et al.* Altered Corticostriatal Functional Connectivity in Obsessive-compulsive Disorder. *Archives of General Psychiatry* **66,** 1189–1200. ISSN: 0003-990X. https://doi.org/10.1001/archgenpsychiatry.2009.152 (2022) (Nov. 2009).
2. Naze, S. *et al.* Mechanisms of imbalanced frontostriatal functional connectivity in obsessive-compulsive disorder. *Brain* **146,** 1322–1327. ISSN: 0006-8950. https://doi.org/10.1093/brain/awac425 (2023) (Apr. 2023).
3. Van Essen, D. C. *et al.* The WU-Minn human connectome project: an overview. *Neuroimage* **80.** Publisher: Elsevier, 62–79 (2013).
4. Swanson, L. W., Sporns, O. & Hahn, J. D. The network organization of rat intrathalamic macroconnections and a comparison with other forebrain divisions. *Proceedings of the National Academy of Sciences* **116.** Publisher: Proceedings of the National Academy of Sciences, 13661–13669. https://www.pnas.org/doi/10.1073/pnas.1905961116 (2025) (July 2019).
5. Van den Heuvel, O. A. *et al.* Brain circuitry of compulsivity. *European Neuropsychopharmacology* **26.** Publisher: Elsevier, 810–827 (2016).
6. Shephard, E. *et al.* Toward a neurocircuit-based taxonomy to guide treatment of obsessive-compulsive disorder. eng. *Molecular Psychiatry* **26,** 4583–4604. ISSN: 1476-5578 (Sept. 2021).
7. Deco, G. *et al.* Resting-state functional connectivity emerges from structurally and dynamically shaped slow linear fluctuations. *Journal of Neuroscience* **33,** 11239–11252 (2013).
8. Clewley, R. Hybrid Models and Biological Model Reduction with PyDSTool. *PLoS Comput Biol* **8,** e1002628. http://dx.doi.org/10.1371/journal.pcbi.1002628 (2015) (Aug. 2012).
9. Strogatz, S. H. *Nonlinear Dynamics And Chaos: With Applications To Physics, Biology, Chemistry, And Engineering* 1 edition. English. ISBN: 978-0-7382-0453-6 (Westview Press, Cambridge, MA, Jan. 2001).
10. Marjoram, P., Molitor, J., Plagnol, V. & Tavaré, S. Markov chain Monte Carlo without likelihoods. *Proceedings of the National Academy of Sciences* **100.** Publisher: Proceedings of the National Academy of Sciences, 15324–15328. https://www.pnas.org/doi/10.1073/pnas.0306899100 (Dec. 2003).
11. Toni, T., Welch, D., Strelkowa, N., Ipsen, A. & Stumpf, M. P. Approximate Bayesian computation scheme for parameter inference and model selection in dynamical systems. *Journal of The Royal Society Interface* **6.** Publisher: Royal Society, 187–202. https://royalsocietypublishing.org/doi/10.1098/rsif.2008.0172 (July 2008).
12. Sisson, S. A., Fan, Y. & Tanaka, M. M. Sequential Monte Carlo without likelihoods. *Proceedings of the National Academy of Sciences* **104.** Publisher: Proceedings of the National Academy of Sciences, 1760–1765. https://www.pnas.org/doi/10.1073/pnas.0607208104 (Feb. 2007).
13. Givens, G. H. & Raftery, A. E. Local Adaptive Importance Sampling for Multivariate Densities with Strong Nonlinear Relationships. *Journal of the American Statistical Association* **91,** 132–141. ISSN: 0162-1459. https://www.tandfonline.com/doi/abs/10.1080/01621459.1996.10476670 (Mar. 1996).
14. Del Moral, P., Doucet, A. & Jasra, A. Sequential Monte Carlo samplers. en. *Journal of the Royal Statistical Society: Series B (Statistical Methodology)* **68,** 411–436. ISSN: 1467-9868. https://onlinelibrary.wiley.com/doi/abs/10.1111/j.1467-9868.2006.00553.x (2006).
15. Duan, J.-C., Li, S. & Xu, Y. Sequential Monte Carlo optimization and statistical inference. en. *WIREs Computational Statistics* **15.** _eprint: https://onlinelibrary.wiley.com/doi/pdf/10.1002/wics.1598, e1598. ISSN: 1939-0068. https://onlinelibrary.wiley.com/doi/abs/10.1002/wics.1598 (2023) (2023).
